# Tropical diabetes hand syndrome with autoamputation of the digits: case report and review of literature

**DOI:** 10.11604/pamj.2014.18.199.3593

**Published:** 2014-07-05

**Authors:** Taiwo Hussean Raimi, Oluwole Ojo Alese

**Affiliations:** 1 Department of Medicine, Ekiti State University Teaching Hospital, Ado-Ekiti, Ekiti State, Nigeria; 2 Department of Family Medicine, Ekiti State University Teaching Hospital, Ado-Ekiti, Ekiti State, Nigeria

**Keywords:** Hand infection, diabetes, outcome

## Abstract

The tropical diabetes hand syndrome is a complication affecting patients with diabetes mellitus in the tropics, and consists of localized cellulitis, swelling and ulceration of the hands which may progress to fulminant sepsis and gangrene of the whole limb. It is associated with a poor outcome. We report a 32 year old woman with tropical diabetes hand infection with autoamputation of the digits, review the relevant literature, and highlight the need for prevention and early hospital presentation in diabetics with hand infection, in order to prevent potentially crippling or fatal complications.

## Introduction

The tropical diabetes hand syndrome is a complication affecting patients with diabetes mellitus in the tropics, and consists of localized cellulitis, swelling, and ulceration of the hands which may progress to fulminant sepsis and gangrene of the whole limb [1]. It is not as common as diabetic foot ulcer but equally carries a poor prognosis [2], and unlike DMFS, peripheral neuropathy and peripheral vascular disease were initially thought not to play a critical role in its development [3].

## Patient and observation

A 32-year old woman diagnosed with diabetes mellitus 2 months before presentation came on self-referral to our center on account of right hand ulcer of three months duration. The ulcer started with pain and later swelling of the ring finger with rapid progression to involve the whole of the hand and forearm which later transform into ulcer affecting the whole of the hand. No history of fever at the time of presentation to our facility. She had 6 month history of polyuria, polydipsia and weight loss. There was no blurring of vision, paraesthesia or recurrent boil. There was no previous delivery of macrosomic babies or fetal wastages. She was not a known patient with hypertension, peptic ulcer disease or bronchial asthma. She had no family history of diabetes. She neither smoke cigarette nor drink alcohol. She was on self-medications including oral ampicillin, cloxacillin, diclofenac, paracetamol and glimeperide.

**Figure 1:**
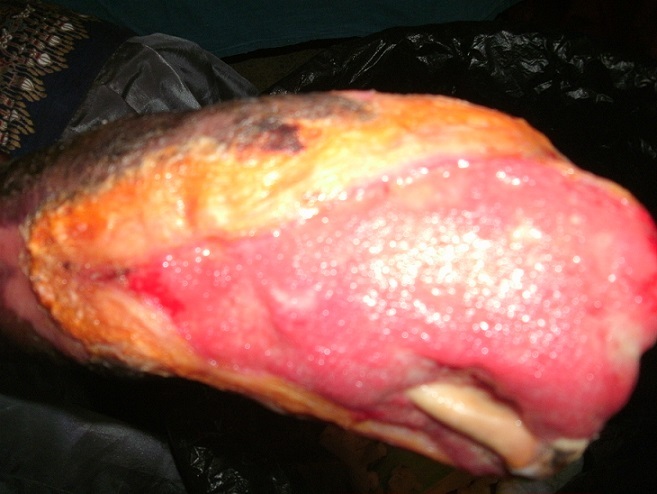
The right hand of the patient at presentation. The fourth proximal phalangeal bone was exposed, and there was auto-amputation of the distal and middle phalanges of all the fingers

She initially managed the ulcer with herbal remedies and later presented to another tertiary health center where she was managed for hyperglycaemic emergency, but left against medical advice (LAMA) after 10days when she was offered amputation of the right hand. Her general examination was normal. Examination of her right hand revealed an extensive ulcer involving both the dorsum and plantar surface of the hand extending to about 2cm above the wrist. The ulcer had granulation tissue with island of slough. The fourth proximal phalangeal bone was exposed, and there was auto-amputation of the distal and middle phalanges of all the fingers (Figure 1). The radial and ulnar pulsation were palpable but of reduced volume. The joint position sense, sensation to light touch and pain were intact. Systemic examination revealed normal findings.

**Figure 2:**
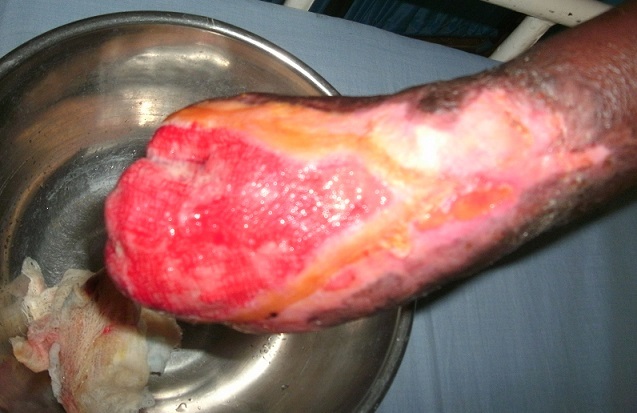
The right hand of the patient before discharge. The wound has healthy granulation tissue, and the phalanx is no longer visible

Laboratory investigation revealed random plasma glucose of 13.5mmo/L, serum sodium of 140.7mmol/L, serum potassium of 4.0mmol/L, serum chloride 111.0mmol/L and serum bicarbonate of 20.7mmol/L. The serum urea and creatinine were 3.59mmol/L and 74µmo/L respectively. Urinalysis revealed glycosuria but there was no proteinuria and ketonuria, while the complete blood count revealed a packed cell volume of 34%, white blood cell count of 14.8 x109/L with neutrophil of 70%, and lymphocyte of 29%. Wound swab for culture yielded *Klebsiella spp*. She was managed with antibiotics, insulin, vitamin C and analgesics. The wound was dressed daily with honey and later with platelet- derived growth factor. She was offered below elbow amputation and wound grafting which she refused (Figure 2). She was discharged six weeks after admission with fasting plasma glucose of 7.4mmol/L before discharge and was followed up at the outpatient department. Her glycosylated heamoglobin six months post discharge was 6.2%. The wound healed seven months after she was discharged.

## Discussion

TDHS encompasses a localized cellulitis with variable swelling and ulceration of the hands to progressive, fulminant hand sepsis, and potentially fatal synergistic form of gangrene. The term was first used in 1998 by Gill et al [1] although the clinical condition was first reported in medical literature in the late seventies [4]. It usually follows minor or unrecognized trauma [3].

The age at presentation is variable. Whereas some reports and studies suggested that older patients were affected [2, 3, 5], others revealed that younger patients in their active life were affected [6]. Our patient was in her prime age. Female preponderance was reported by some authors [7, 8, 9], while others found a higher male to female ratio in their study [2, 6]. The predominant gender appear to be dependent on the geographical location: more women in sub-Sahara Africa, where women are more involved in manual and domestic work including fishing, which exposes them to frequent hand trauma; more men in Asia where males are the main labor force. Nevertheless, a study of 72 patients with diabetic hand infections in Tanzania revealed an equal gender representation [3]. Most of the reported cases of TDHS occurred in people with established diabetes, typically T2DM [1, 2, 8, 9, 10] whereas in our patient, it was the presenting disease. In a case series, 16 out of 48 patients (33.3%) had undiagnosed diabetes mellitus and majority had type1DM [6].

Our patient had peripheral vascular disease but had no features suggestive of peripheral neuropathy. Although an earlier study [3] suggested that neuropathy and peripheral vascular disease were not significant in the development of TDHS, the first analytical study on TDHS revealed that independent risk factors for TDHS include neuropathy in addition to poorly controlled diabetes, insulin treatment, or malnutrition [11]. Similarly, more recent studies [2, 9] found that they were important risk factors. In most cases, the immediate precipitating event in TDHS is trauma which may be trivial [1, 2, 6, 8]. The patient presented by us had no history of trauma, similar to reports by some authors [12, 13, 14]. Other features of TDHS include poor glycemic control, delayed presentation, severe deep palmar infection, high risk of amputation and mortality. As in the case presented, delayed presentation and poor glycemic control were consistent findings in previous reports and was usually due to ignorance, home treatment or management by traditional healer [1, 2, 6, 8, 9, 13]. The patient presented to the first hospital in a state of coma, and to us when she had already developed autoamputation of the fingers and in poor glycemic state.

The Management of TDHS should be prompt and aggressive in order to prevent crippling deformity or death. Patients should be hospitalized and placed on intravenous antibiotics, insulin and the wound debrided or abcess incised and drained [2, 11, 13]. Our patient responded to insulin, antibiotics and local wound dressing but ended up with deformity. Most patients with TDHS would require surgical intervention and a significant number may end up with amputation. For example 100% surgical intervention rate was reported by Wang et al. [2] and Unachukwu et al. [8]; 96% intervention rate with 12% amputation by Kour et al [15]; 50% intervention rate with 9.7% amputation by Abbas et al. [3]Chaurbry et al. [6], Mann et al. [4], and Faraoun et al. [9], also reported a 62.5%,35%, and 23.1% amputation rate respectively. These outcomes reflect the delay in presentation. Our patient had auto-amputation of all the digits of the affected hand rather than surgical amputation. This is rare in the literature. Nevertheless, the optimal management of her case still requires surgical intervention, which she declined.

Mortality in TDHS is high and ranged from 19.2% to 100% in some of the older studies [8, 9, 16]. However, outcome has improved (0%-1.7%) in more recent studies [2, 6]. Most of the recent case reports also revealed zero mortality [10, 12, 14]. Our patient survived, but ended up with deformity.

## Conclusion

The outcome of tropical diabetes hand infection is poor. Structured education on hand care in patients with diabetes should be incorporated into diabetes care, and early hospital presentation should be encouraged in diabetics with hand infection.
